# The quest for a biological phenotype of adolescent non-suicidal self-injury: a machine-learning approach

**DOI:** 10.1038/s41398-024-02776-4

**Published:** 2024-01-25

**Authors:** Ines Mürner-Lavanchy, Julian Koenig, Corinna Reichl, Johannes Josi, Marialuisa Cavelti, Michael Kaess

**Affiliations:** 1 University Hospital of Child and Adolescent Psychiatry and Psychotherapy, University of Bern, Bern, Switzerland; 2 Department of Child and Adolescent Psychiatry, Psychosomatics and Psychotherapy, Faculty of Medicine and University Hospital Cologne, University of Cologne, Cologne, Germany; 3 Department of Child and Adolescent Psychiatry, Centre for Psychosocial Medicine, University of Heidelberg, Heidelberg, Germany

**Keywords:** Predictive markers, Physiology, Diagnostic markers, Human behaviour

## Abstract

Non-suicidal self-injury (NSSI) is a transdiagnostic psychiatric symptom with high prevalence and relevance in child and adolescent psychiatry. Therefore, it is of great interest to identify a biological phenotype associated with NSSI. The aim of the present study was to cross-sectionally investigate patterns of biological markers underlying NSSI and associated psychopathology in a sample of female patients and healthy controls. Comprehensive clinical data, saliva and blood samples, heart rate variability and pain sensitivity, were collected in *n* = 149 patients with NSSI and n = 40 healthy participants. Using machine-based learning, we tested the extent to which oxytocin, dehydroepiandrosterone (DHEA), beta-endorphin, free triiodothyronine (fT3), leukocytes, heart rate variability and pain sensitivity were able to classify participants regarding their clinical outcomes in NSSI, depression and borderline personality disorder symptomatology. We evaluated the predictive performance of several models (linear and logistic regression, elastic net regression, random forests, gradient boosted trees) using repeated cross-validation. With NSSI as an outcome variable, both logistic regression and machine learning models showed moderate predictive performance (Area under the Receiver Operating Characteristic Curve between 0.67 and 0.69). Predictors with the highest predictive power were low oxytocin (OR = 0.55; *p* = 0.002), low pain sensitivity (OR = 1.15; *p* = 0.021), and high leukocytes (OR = 1.67; *p* = 0.015). For the psychopathological outcome variables, i.e., depression and borderline personality disorder symptomatology, models including the biological variables performed not better than the null model. A combination of hormonal and inflammatory markers, as well as pain sensitivity, were able to discriminate between participants with and without NSSI disorder. Based on this dataset, however, complex machine learning models were not able to detect non-linear patterns of associations between the biological markers. These findings need replication and future research will reveal the extent to which the respective biomarkers are useful for longitudinal prediction of clinical outcomes or treatment response.

## Introduction

Non-suicidal self-injury (NSSI) denotes self-injurious behavior in the absence of suicidal intent (American Psychiatric Association, 2013), is highly prevalent in adolescents from the general population [1] as well as in clinical samples [2] and has been suggested as a transdiagnostic risk marker of psychopathology [3]. NSSI results from a complex interaction of psychological, social and biological mechanisms. Research on the biological factors contributing to the development and maintenance of NSSI is in its infancy. A recently advanced biological model of NSSI distinguishes distal from proximal biological traits as well as biological states [4]. While distal biological traits may include genetic, epigenetic or perinatal and early-life vulnerabilities increasing predisposition for NSSI, more proximal biological traits include moderately stable alterations in brain-circuitry, the stress response systems and pain processing, enhancing the risk for NSSI.

Given that one of the main functions of NSSI is the regulation of unpleasant emotions and interpersonal experiences [5], the biological stress response systems may play a central role in NSSI. While evidence on alterations in hypothalamic-pituitary-adrenal (HPA) axis functioning in adolescents with NSSI is accumulating [6–11], a related neuroendocrine system that also responds to stress, the hypothalamic-pituitary-thyroid (HPT) axis, has received far less attention within NSSI research. A first study found blunted free triiodothyronine (fT3)/ free thyroxine (fT4) ratios in adolescents engaging in NSSI compared to healthy controls [12], which might indicate altered HPT axis functioning in youths with NSSI. A neurosteroid involved in the production of stress hormones is dehydroepiandrosterone (DHEA) [13]. Although its role for NSSI has to our knowledge not been reported so far, one study showed reduced levels of DHEA in combat veterans with vs. without a history of suicide attempt [14]. Reduced DHEA may lead to an increased vulnerability to the neurotoxic effects of stress [15, 16] and stress-related psychopathology, in turn, is associated with NSSI. Another more prominent peripheral stress response system is the autonomic nervous system (ANS). Existing evidence to date suggests reduced vagally-mediated heart rate variability (HRV) in individuals with NSSI, presumably as a function of underlying personality pathology [17–19].

Per definition, NSSI involves the destruction of one’s own body tissue, which is usually accompanied by a physical sensation, and, arguably pain. The pain analgesia hypothesis suggests that youths with NSSI feel little to no pain [20], which lends them vulnerable to engage in the behavior. Meta-analytic research has confirmed lower pain sensitivity, a higher pain threshold, and tolerance in adolescents with NSSI history compared to healthy controls [21]. These alterations in pain perception suggest that endogenous opioids implicated in the regulation of pain may play a role in the emergence and maintenance of NSSI. Opioid deficiency theories suggest that lower resting levels of beta endorphin (BE) lead individuals to restore homeostasis by engaging in NSSI, which likely results in releases of BE [22]. Such hypotheses have been empirically substantiated in adult [23] and adolescent NSSI [24] with the latter study showing lower levels of BE immediately before compared to after experimentally induced pain. Therefore, BE might be an important biological marker of NSSI.

Another neuropeptide of potential relevance for NSSI is oxytocin, which is involved in regulating social interaction, and has received attention in the context of suicidality and suicidal behavior as well as Borderline Personality Disorder (BPD). Suicide attempters had lower oxytocin concentrations in the cerebrospinal fluid [25, 26] and serum [27] indicating the importance of social predictors of suicidal behaviors [28]. Reduced plasma oxytocin has further been associated with the severity of BPD pathology, an association which was mediated by adverse childhood experiences [29]. This finding suggests that childhood adversity contributes to alterations in the oxytocin system in patients with BPD. While NSSI is a core, but non-pathognomic symptom of adolescent BPD, the role of oxytocin has yet to be further examined in the context of NSSI.

Recent studies focus on the predictive role of inflammatory markers known to interact with neuroendocrine processes. Driven by findings linking inflammation with child maltreatment [30, 31], and depression [32], a recent investigation found an increased number of leukocytes and leukocyte/cortisol ratio in adolescents with NSSI compared to healthy controls [33]. The inflammatory markers further correlated with depressive symptoms across study groups, indicating an interaction between affective symptomatology and inflammatory processes.

Taken together, there is emerging evidence for the role of proximal biological markers in NSSI. Each of the above-mentioned biological markers has previously been associated with NSSI in the sample used for the present investigation [12, 24, 33–36]. However, in the quest for a biological phenotype associated with NSSI, the *combination* of several markers is necessary. In that respect, the use of flexible statistical machine learning models allows for the detection of non-linear patterns of associations (i.e., relationships described by quadratic or higher polynomial terms; a specific combination of, or interaction between two or more biological markers; or a particular ratio between several biomarkers) between biological markers and NSSI. Therefore, the aim of the present study was to use complex machine learning algorithms to investigate whether several proximal biological trait markers of the neuroendocrine, autonomic nervous and pain systems could serve to detect a biological phenotype associated with NSSI and depression severity as well as BPD symptomatology in a clinical sample of adolescents.

## Materials and methods

### Participants and procedure

Data collection for the present study was nested in a cohort study of consecutively recruited adolescents seeking help at the outpatient clinic for adolescent risk-taking and self-harming behavior (AtR!Sk; Ambulanz für Risikoverhalten und Selbstschädigung) [35] at the Clinic for Child and Adolescent Psychiatry, University Hospital Heidelberg (ethical approval: S-449/2013). Within the cohort study, patients underwent comprehensive clinical diagnostic assessments performed by trained clinicians and were subsequently invited to participate in a second appointment with an extensive neurobiological assessment (AtR!Sk-Bio study; ethical approval: S-514/2015). General inclusion criteria were written informed consent of adolescents and their caregivers, age between 12 and 17 years and fluent German language. Exclusion criteria were acute psychosis, pregnancy, and neurological, endocrinological, or cardiovascular primary diseases. Healthy controls were recruited via advertisement. Exclusion criteria for controls were the same as for the patient group. Further exclusion criteria for the control group only were lifetime self-harming behavior, lifetime psychological or psychiatric treatment, or any current psychiatric disorder. All participants received an allowance of 40€ for study participation. For the present study, data from all female participants who took part in the neurobiological assessment and only data from patients reporting at least one day with NSSI was eligible.

### Clinical assessment

Information obtained during the clinical assessment included sex, date of birth, medication, smoking and drug use. In patients, outcome variables for the present study were assessed using semi-structured interviews: NSSI preceding the interview was assessed using the German version of the Self-Injurious Thoughts and Behaviors interview (SITBI-G) [37, 38]. The variable of interest was whether a patient had engaged in NSSI at least five times during the year before the interview (yes/no), which is coherent with criterion A of NSSI disorder in the Diagnostic and Statistical Manual of Mental Disorders (DSM-5). The number of symptoms met for Borderline Personality Disorder, was obtained using the respective section of the Structured Clinical Interview for DSM-IV Personality Disorders (SCID-II) [39]. To assess potentially comorbid psychiatric diagnoses, the Mini-International Neuropsychiatric Interview for Children and Adolescents (M.I.N.I.-KID 6.0) [40] was used. Further, in patients and healthy controls, depressive symptoms were ascertained by the German version of the Depression Inventory for Children and Adolescents questionnaire (DIKJ) [41].

Healthy controls underwent a telephone screening before their first assessment to ensure that they had no history of NSSI or suicidal behavior. At the clinic, they then participated in a shortened clinical interview to ascertain they did not meet the criteria for any current mental disorder and were not under psychological or pharmacological treatment. Whenever healthy controls reported any symptoms indicative of the presence of a psychiatric disorder, the M.I.N.I.-KID was used as an additional diagnostic tool. Those participants meeting the criteria for any psychiatric disorder in an additional diagnostic interview were compensated for their participation in the diagnostic assessment and excluded from further study appointments.

### Biological measures

Biological measures obtained included oxytocin, DHEA, beta-endorphin, fT3, leukocytes, heart rate variability and pain sensitivity. Stable markers of cortisol secretion, such as diurnal cortisol, cortisol awakening response or cumulative measures were not collected as a part of the neurobiological assessment and therefore not included in the present investigation.

#### Blood samples

Fasting peripheral blood samples were taken between 8:30 and 9:00 a.m. to control for diurnal variation in hormone levels [24]. Samples were analyzed at the central laboratories of Heidelberg University Hospital according to accredited routines. FT3 levels were determined by an ADVIA Centaur immunoassay, according to the protocol of the manufacturer. BE and oxytocin levels were ascertained using an enzyme-linked immunosorbent assay (ELISA) by Cloud Clone (Houston, TX, USA) and DHEA levels were determined using an in-house radioimmunoassay. Reference ranges were the following: fT3 2.0–4.2 ng/l; BE: 20,5–121,6 pg/ml; oxytocin: 70–165 pg/ml; DHEA: 1,3–4 μg/ml. Leukocytes were measured using flow cytometry (ADVIA2120®, Siemens Healthineers).

#### HRV

ECG data were recorded continuously as inter-beat-interval (IBI) at 1024 Hz using an ECGMove 3 sensor (movisens GmbH, Karlsruhe, Germany). The ECG signal was recorded during a 5 min baseline period while patients were engaged in a minimally demanding color detection task (CDT; Jennings et al [42]). ECG data were processed in Kubios HRV 3.0 Premium [43, 44]. R-Peak detection was manually corrected, and artifacts were quantified and removed. Smoothing priors was selected as a detrending method (λ 500) for IBI data and Kubios output was exported for subsequent analysis of HRV using the R Heart Rate Variability package (RHRV) [45]. The root mean square of successive differences (RMSSD) of IBIs (in ms) was extracted and used as a time-domain measure of vmHRV reflecting cardiac vagal activity [46].

#### Pain sensitivity

Pain sensitivity was assessed using an AHP-1800CPV Versatile Cold/Hot Plate (TECA Corp., Chicago, IL, USA) and a predefined programmed sequence for the temperature. Baseline adaptation temperature was set at 32 °C. Temperature was sealed off at 50 °C to avoid tissue damage. Participants were instructed to place their non-dominant hand flat on the plate as soon as baseline temperature was reached. After a 3-min adaptation phase, temperature raised up to 50 °C within 4 min, increasing linearly by 1 °C across 13.3 s. Participants were asked to keep their hand firmly on the plate until the pain became intolerable. Pain threshold was reached at the first pain sensation and was measured in °C.

### Statistical analyses

Before building the machine-learning models, data was cleaned and prepared for analyses. Patients with missing data for one of the predictors were excluded from the analyses. Predictors were not transformed or standardized prior to model fitting since models using classification and regression trees are not sensitive to predictor scaling or transformation. Finally, predictor variables were standardized to allow for comparison of statistical coefficients. Predictors considered for all models were fT3, BE, oxytocin, DHEA, leukocytes, HRV and pain sensitivity. Three outcomes were tested: NSSI in the past year (<= 5 times; yes/no), depression (continuous) and number of BPD criteria fulfilled (0–9 treated as continuous variable).

For each outcome, four models were fit: linear or logistic regression (for continuous and binary outcomes, respectively); elastic net regression [47]; random forests [48]; and gradient boosted trees [49]. These models were selected since they are among the most well-established models for supervised learning [50]. Elastic nets build upon linear and logistic regression, but include a regularization term to perform variable selection and shrink coefficients to improve generalization to unseen data. Random forests and gradient boosted trees are more flexible models without additivity or linearity assumptions and allowing arbitrary interactions between the predictors.

Since the performance of a predictive model on the data used for training is typically overestimating the performance one would see on unseen data [51], it is recommended to internally validate prediction models using techniques such as bootstrapping or repeated cross-validation [52]. We internally validated each model using repeated cross-validation with five folds and twenty repetitions. For the binary outcomes, the metric considered was the area under the Receiver Operator Characteristic (ROC) curve (AUC). For the continuous outcomes, the metrics used were the root mean squared error (RMSE) and the mean absolute error (MAE). For each metric, we report the mean and standard deviation across all folds of the repeated cross-validation.

Model fitting and evaluation was performed in R Statistical Software (v4.2.2 [53]). Cross-validation of all models was done using the caret package (v6.0 [54]). The elastic net regression models were fit using the glmnet package (v4.1 [55]). Random forests were fit using the ranger package (v0.14.1 [56]), and gradient boosted trees were fit using the xgboost package (v1.7.3.1; Chen & Guestrin, [49]). Analysis code can be made available upon request from the corresponding author.

## Results

### Demographic and clinical patient characteristics

Of *n* = 242 patients and *n* = 49 healthy controls enrolled in the AtR!Sk-Bio study, data from *n* = 42 male patients and *n* = 2 healthy male controls were excluded. Moreover, n = 9 patients were excluded due to reporting zero days with NSSI incidents in the past year. Further, data from patients with missings in one of the predictors were excluded, in this order: leukocytes, *n* = 17 patients and *n* = 3 controls; ft3, *n* = 3 patients; oxytocin *n* = 11 patients and *n* = 4 controls; BE *n* = 1 patient; pain sensitivity *n* = 2 patients; HRV *n* = 7 patients; DHEA *n* = 1 patient. The final study sample therefore comprised *n* = 149 patients and *n* = 40 healthy controls (Table 1). While groups were similar in age, a larger proportion of patients reported taking psychoactive medication, smoking, and drug consumption. As expected, patients reported more days with NSSI incidents in the past year, had a higher number of criteria fulfilled for BPD and a higher depression severity.

**Table 1:** Sample characteristics.

	Controls (*N* = 40)	Patients (*N* = 149)	Overall (*N* = 189)
*Age*			
Mean (SD)	14.8 (1.28)	14.9 (1.48)	14.9 (1.44)
Median [Min, Max]	15.0 [12.0, 17.0]	15.0 [12.0, 19.0]	15.0 [12.0, 19.0]
*Psychoactive medication*			
Yes	0 (0%)	13 (8.7%)	13 (6.9%)
No	40 (100%)	136 (91.3%)	176 (93.1%)
*Drug consumption*			
Yes	7 (17.5%)	44 (29.5%)	51 (27.0%)
No	33 (82.5%)	105 (70.5%)	138 (73.0%)
*Smoking*			
Yes	9 (22.5%)	76 (51.0%)	85 (45.0%)
No	31 (77.5%)	73 (49.0%)	104 (55.0%)
*fT3 [ng/l]*			
Mean (SD)	3.55 (0.451)	3.40 (0.378)	3.43 (0.398)
Median [Min, Max]	3.50 [2.59, 4.40]	3.38 [2.35, 4.67]	3.38 [2.35, 4.67]
*Leukocytes [/nl]*			
Mean (SD)	5.63 (1.49)	6.63 (1.87)	6.42 (1.84)
Median [Min, Max]	5.38 [3.14, 9.31]	6.33 [3.43, 14.5]	6.17 [3.14, 14.5]
*DHEA-S [µg/ml]*			
Mean (SD)	6.78 (31.2)	1.96 (1.07)	2.98 (14.4)
Median [Min, Max]	1.66 [0.600, 199]	1.68 [0.390, 5.79]	1.68 [0.390, 199]
*Oxytocin [µg/ml]*			
Mean (SD)	211 (131)	135 (147)	151 (147)
Median [Min, Max]	181 [64.3, 795]	106 [40.4, 1540]	131 [40.4, 1540]
*Beta-endorphin [µg/ml]*			
Mean (SD)	38.4 (20.1)	34.4 (28.2)	35.3 (26.7)
Median [Min, Max]	39.2 [2.40, 94.3]	26.0 [0.400, 224]	28.6 [0.400, 224]
*Pain threshold* [C°]			
Mean (SD)	41.7 (3.28)	42.7 (3.80)	42.5 (3.71)
Median [Min, Max]	42.0 [34.3, 46.9]	43.5 [34.1, 50.0]	43.0 [34.1, 50.0]
*vmHRV*			
Mean (SD)	58.4 (28.6)	54.9 (28.6)	55.6 (28.5)
Median [Min, Max]	51.3 [21.4, 132]	48.1 [13.6, 178]	49.1 [13.6, 178]
*No. days with NSSI events past year*			
Mean (SD)	0 (0)	56.9 (66.9)	44.7 (63.7)
Median [Min, Max]	0 [0, 0]	30.0 [1.00, 350]	18.0 [0, 350]
*No. of fulfilled criteria for BPD*			
Mean (SD)	0.0750 (0.350)	3.24 (2.09)	2.57 (2.27)
Median [Min, Max]	0 [0, 2.00]	3.00 [0, 8.00]	2.00 [0, 8.00]
*Depression severity (DIKJ raw score)*			
Mean (SD)	6.33 (5.23)	29.3 (9.04)	24.0 (12.8)
Median [Min, Max]	5.00 [0, 29.0]	30.0 [7.00, 47.0]	28.0 [0, 47.0]
Missing	1 (2.5%)	19 (12.8%)	20 (10.6%)
*Diagnoses*^*a*^			
F10-F19, *n (%)*	–	24 (16.1)	
F30-F39, *n (%)*	–	92 (61.7)	
F40-F48, *n (%)*	–	65 (43.6)	
F50-F59, *n (%)*	–	19 (12.8)	
F60-F69, *n (%)*	–	54 (36.2)	
F80-F89, *n (%)*	–	1 (0.7)	
F90-F98, *n (%)*	–	32 (21.5)	

*SD* standard deviation, Smoking: on more than one day in the last year. Drug consumption: at least one day of drug consumption during the last year. *fT3* free triiodothyronine, *DHEA* dehydroepiandrosterone, *vmHRV* vagally mediated heart rate variability, *NSSI* non-suicidal self-injury, *BPD* borderline personality disorder, *DIKJ* Depressionsinventar für Kinder und Jugendliche.

^a^no F0, F2, or F7 disorders were diagnosed.

### Association between biomarkers and clinical outcome

**Fig. 1:**
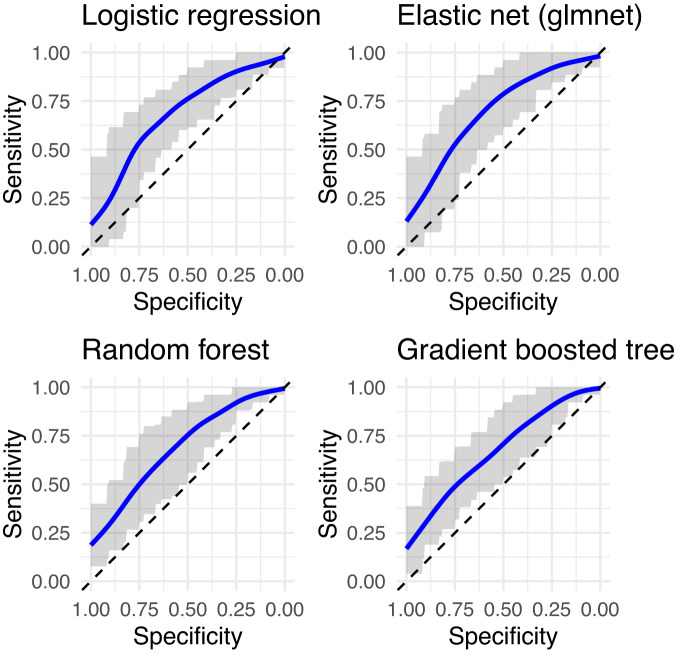
ROC curves for models predicting NSSI < or ≥ 5 in the last year. The blue line is the smoothed average of the ROC curves over all folds, the gray band covers 80% of the sensitivity values across all folds at each level of specificity. The dashed diagonal line corresponds to a useless predictor (chance). The more above and to the left of the diagonal, the better the predictor.

All models had moderate strength in predicting who did and did not engage in NSSI at least five times during the past year (Table 2 and Fig. 1). Indices of model accuracy (ROC and ROCSD) were similar across the different models, with no evidence that machine learning models (elastic net, random forest, gradient boosted tree) outperformed conventional logistic regression. Across all repetitions of the cross-validation (five folds and 20 repetitions), on average, 87.8% of the sample were predicted to belong to the NSSI group in logistic regression (94.1% in elastic net regression, 84.6% in random forest and 79.8% in gradient boosted tree models).

**Table 2:** Receiver operating characteristics and standard deviations of models predicting NSSI compared in cross-validation.

Model	ROC	ROCSD
Logistic regression	0.686	0.085
Elastic net	0.700	0.085
Random forest	0.684	0.076
Gradient boosted tree	0.671	0.079

*ROC* receiver operating characteristics, *ROCSD* standard deviation of ROC.

In the logistic regression, predictors with the highest predictive power were oxytocin, leukocytes and pain sensitivity (Table 3). Within this model, lower oxytocin levels, a higher number of leukocytes and lower pain sensitivity (i.e., higher pain threshold) were associated with the presence of NSSI in the past year.

**Table 3:** Logistic regression for NSSI in the past year (<= 5 times; yes/no).

Predictors	OR	CI	p
(Intercept)	2.49	1.76–3.57	0.100
fT3 [ng/l]	1.03	0.73–1.46	0.858
Leukocytes [l/nl]	1.67	1.13–2.57	0.015
DHEA-S [µg/ml]	0.76	0.00–1.11	0.433
Oxytocin [µg/ml]	0.55	0.37–0.78	0.002
Beta-endorphin [µg/ml]	0.90	0.64–1.28	0.549
Pain threshold [C°]	1.51	1.07–2.15	0.021
vmHRV	0.89	0.64–1.25	0.492

*OR* odds ratio, *fT3* free triiodothyronine, *DHEA* dehydroepiandrosterone, *vmHRV* vagally mediated heart rate variability, *NSSI* non-suicidal self-injury. Tjur *R*^2^ = 0.141.

For the other two outcomes, i.e., number of BPD criteria and depression symptoms, models including the biological variables were not better than the null models (predicting the average outcome), indicating that the biological variables did not have any predictive value.

## Discussion

Based on the biological markers included, both logistic regression and machine learning models showed moderate accuracy to predict, whether individuals engaged in NSSI more or less than five times during the past year. Out of the seven biological variables, oxytocin, leukocytes and pain sensitivity had the highest predictive power, suggesting that lower levels of oxytocin and lower pain sensitivity, as well as a higher number of leukocytes were associated with engagement in NSSI. This finding is in line with previous studies investigating associations between oxytocin, leukocytes and pain sensitivity and NSSI separately [21, 26, 33]. However, the biological markers did not serve to predict BPD symptom severity or depression.

With combining different biological markers into one analysis, we aimed to leverage machine learning to detect a biological phenotype for NSSI in a sample of help-seeking adolescents. In principle, flexible machine learning models allow for the detection of complex non-linear patterns of associations. E.g., it is thinkable, that the relationship between a certain biological variable and clinical outcome can be described by a quadratic or even more complex function. Even more likely, specific (non-linear) interactions or a particular ratio between two or more biological markers might be predictive of NSSI. However, in our sample, the additional flexibility of the more complex machine learning models had no advantage over the simpler logistic regression. It is possible, that in larger patient samples, complex models could detect such non-linear patterns with higher predictive power for NSSI than logistic regression. In this sense, it would be helpful to routinely obtain biological data as a part of in- and outpatient treatment via biobanks. Even in very large samples, however, the predictive accuracy and the clinical relevance of such models remains to be determined.

We acknowledge that other biological variables not examined in the present study, might be valuable candidates for a biological phenotype of NSSI. As such, altered HPA axis functioning has consistently been found in adolescent NSSI [6–10]. On the level of the central nervous system, patients with NSSI showed lower prefrontal cortex oxygenation [57], decreased brain volume in regions associated with emotion regulation [58], and worse neural integration and efficiency [59]. Further potential biological markers with limited evidence so far, include genetic polymorphisms related to serotonergic neurotransmission [60].

Several limitations should be considered when interpreting the findings of our study. First, the characteristics of our sample are somewhat confounded with the outcome of NSSI studied. The data set consisted of patients who engaged in NSSI at least once during the past year as well as healthy control participants. Therefore, the group variable (patients vs. controls) is correlated with the outcome variable, i.e., more or less than 5 incidents of NSSI during the past year. Consequently, predicting the NSSI outcome in our dataset was probably easier than predicting this outcome in a pure patient sample. This might also have contributed to the fact that models did not show accuracy in predicting the severity of BPD or depression in our sample of adolescents engaging in NSSI. Second, we investigated biological characteristics in cross-sectional data. Given the developmental trajectories of NSSI in the function of age, duration and severity of illness (e.g. ref. [61]), longitudinal studies are required to determine whether there are defined biological substrates underlying NSSI merely varying in degree of severity along a continuum or whether the associated biological characteristics differ substantially depending on the progression of disease (e.g., in young individuals at the onset of mental disorder vs. older patients with long-standing psychopathology, such as BPD). Moreover, only longitudinal designs allow to examine whether neurobiological markers or, finally, a biological phenotype serves to predict clinical outcome, course of illness or therapy response. A further important limitation of our study is that we did not include a clinical control group without any NSSI and all patients were recruited through the outpatient clinic for risk-taking and self-harm behavior. Therefore, the specificity of our findings to NSSI remains to be established. However, there were *n* = 24 patients with 1 to 4 days with NSSI in the last year, i.e., not fulfilling the DSM-5 criteria for NSSI disorder. Finally, we did not include male adolescents with NSSI. Hence, we were unable to identify the potential influence of sex on our findings.

To conclude, we found a biological phenotype associated with NSSI that mainly consisted of low oxytocin levels, high leukocytes and reduced pain sensitivity. While our study adds to the increasing evidence of biological markers for NSSI, we were unable to find specific non-linear patterns of associations between several markers. In larger datasets, flexible machine learning models might be able to detect more complex patterns of associations possibly delineating a biological phenotype underlying adolescent NSSI.

## Data Availability

Due to the nature of this research project, participants did not provide consent for their data to be shared publicly, so supporting data is not publicly available. However, anonymized data can be made available upon request from the corresponding author.
